# Quantitative Characterization of Cantharidin in the False Blister Beetle, *Oedemera podagrariae ventralis*, of the Southern Slopes of Mount Elborz, Iran

**DOI:** 10.1673/031.012.15201

**Published:** 2012-12-28

**Authors:** S.M. Abtahi, M.R. Nikbakhtzadeh, H. Vatandoost, A. Mehdinia, A. Rahimi-Foroshani, M. Shayeghi

**Affiliations:** ^1^ Department of Medical Entomology and Vector Control, School of Public Health, Tehran University of Medical Sciences, Tehran, Iran; ^2^ Department of EEOB, 300 Aronoff Laboratory, 318 W 12th Avenue, Ohio State University, Columbus, OH 43210, USA; ^3^ Department of Marine Living Resources, Iranian National Institute for Oceanography, Tehran, Iran; ^4^ Department of Epidemiology and Biostatistics, School of Public Health, Tehran University of Medical Sciences, Tehran, Iran

**Keywords:** Oedemeridae

## Abstract

Cantharidin, a potent vesicant and antifeedant agent, is produced by two families of beetles, Meloidae and Oedemeridae (Coleoptera). In this study, the cantharidin content of oedemerid beetles of central Iran was investigated using the GC-MS method. Cantharidin in both sexes of *Oedemera podagrariae ventralis* Meïneïtrieãs (Oedemeridae) was found in an average of 3.89 µg/beetle in males and 21.68 µg/beetle in females, which are amounts sufficient to irritate human skin. The average of cantharidin in virgin and coupled beetles was 1.35 and 1.62 (µg cantharidin/mg of beetle) respectively. Females had five to six times more cantharidin in their bodies than males, but there was no significant difference between the amount of cantharidin in virgin and coupled females. The results of this study revealed the production of cantharidin in both sexes of beetle.

## Introduction

**Figure 1.**
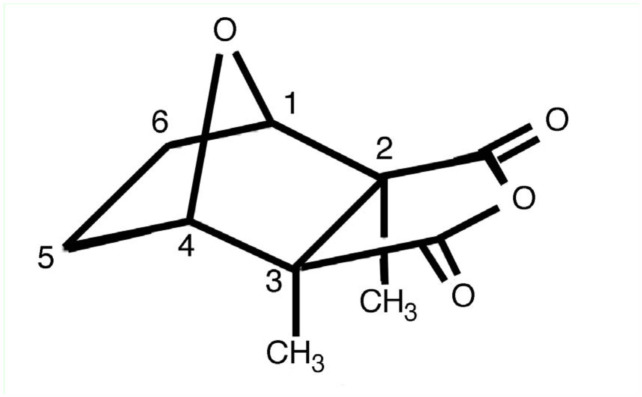
Chemical structure of C_10_H_12_O_4_. High quality figures are available online.

Cantharidin, C_10_H_12_O_4_ (Figure 1), has an important role in the ecology of some insects that use or produce it as a defense mechanism (Carrel and Eisner 1974). Only two families of beetles, Meloidae and Oedemeridae (Coleoptera), are recognized as cantharidin producers in the animal kingdom. The main function of cantharidin in these beetles is to preserve their eggs from predators (Capinera 2008; Mullen and Durden 2009). Cantharidin can cause severe skin blisters, especially when the insects discharge it from their junctions as a defense system or get crushed on a human body (Mizota 2001; Arnett and Thomas et al. 2002; Nikbakhtzadeh and Tirgari 2008).

The Oedemeridae family consists of about 100 genera and 1500 species. The species are distributed worldwide, and so are meloid beetles (Vazquez 2002). There are a few reports about the existence of some oedemerid species in Iran (Svihla 1999, 2006, 2007), but there is no information about their cantharidin content. In this study, the presence of cantharidin was recorded and quantified in the dominant species in the southern slopes of the Elborz Mountains, part of the central region of Iran.

## Materials and Methods

### Beetles

Oedemerids are pollen-feeding insects (Vazquez 2002); therefore, they were collected by hand on flowers during May–June 2009. Beetles were transported alive to the laboratory, and 30 adult beetles were placed in two groups based on sex by investigation of terminal genitalia (15 male and 15 female). The virginity of beetles was determined by investigation of the internal reproductive system.

### Chemical analysis

**Extraction.** All samples were put into small, fused, test tubes separately and freeze-dried for 24 hr at -50° C and 9 × 10^-2^ mbar pressure using a LYOTRAP PLUS freeze-dryer (Scientific Laboratory Supplies Ltd., http://www.scientificlabs.co.uk/) to determine the dry weight. Whole, dried bodies were hydrolyzed in fused test tubes with 200 µl 6 N hydrochloric acid (Technical HCl, 37%, MERCK, http://www.merck.com/) at 120° C for 4 hr to dissolve all body structures and to free bound cantharidines. Subsequently, an equal volume of chloroform was added, each vial was vigorously shaken on a Vortex mixer for 60 sec, and samples were centrifuged (ROTO FIX 32A centrifuge, Hettich, http://www.hettichlab.com/) at 3000 × g for 5 min. Using a Pasteur pipette, the organic phase was filtered and transferred into vials (Holz and Strell 1994; Mebs and Pogoda et al. 2009). All test tubes and vials used had been already silanized for 24 hr by dimethyldichlorosilane solution (CH3)_2_SiCl2, (MERCK).

**GC-MS.** The samples were injected in splitless mode via a 1 µl Hamilton syringe into a 6890N (Agilent, http://www.agilent.com/) gas Chromatograph equipped with a HP-5 (5% phenyl, 95% methylpolysiloxane, non-polar) fused silica capillary column (30 m, ID 0.25 mm ID, 0.33 µm film thickness) that in turn connected to a 5973 mass selective detector (Agilent). The chromatographic conditions were as follows: initial temperature 60° C, temperature increased to 275° C at 10° C/min, an isotherm period of 10 min in 275° C, and then cooled down to 60° C. Helium was used as a carrier gas at a constant flow of 2 mL/min. The electron Impact Ionization (EI 70 ev) provided mass spectra with a characteristic fragmentation of cantharidin: two base peaks of fragments were m/*z* 128 and 96 (M+: 196). The total mass Spectra analyzed by MSD chemstation software and base peaks were compared by the Wiley 275 registry of mass spectral data bank. The quantization of cantharidin was performed using a standard regression curve, drawn by injections of ascending concentrations of authentic cantharidin (Aktin Chemical, Inc., http://www.aktinchemicals.com/) from 0.01 to 250 ppm with y = 61507x - 3E + 06 formula (R^2^ = 0.994).

## Results

**Figure 2.**
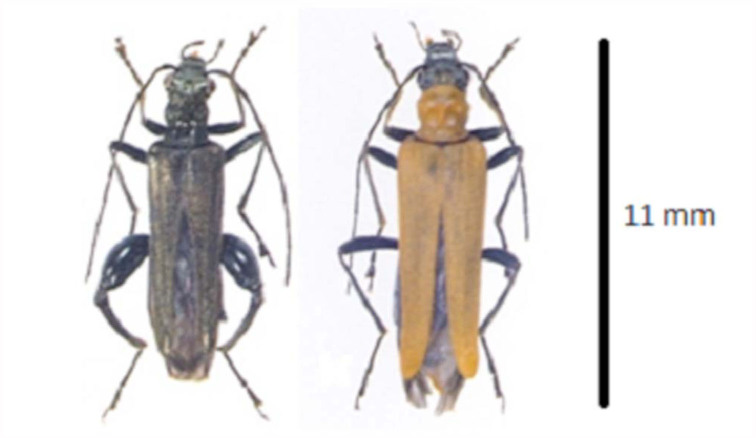
Male (Left) and female (right) of *Oedemera podagrariae ventralis* Meïneïtrieãs. High quality figures are available online.

All the collected samples were *Oedemera podagrariae* ventralis Meïneïtrieãs (Coleoptera: Oedemeridae) based on a related key (Svihla 1999). Cantharidin was detected in both sexes. It had a peak in the chromatogram with the retention time of 12.8 and mass spectra with base peaks at *m/z* 128 and 96. Despite the obvious sexual dimorphism (Figure 2), there was no significant difference between the weights of both sexes (*p* = 0.972). However, intraspecific comparisons showed that females had five to six times more cantharidin in their bodies than males. Quantitative measurements are presented in Table 1. Three females were virgin, but there was no significant difference between the cantharidin amount in coupled and virgin females (*p* = 0.478) (Table 2).

**Table 1.**
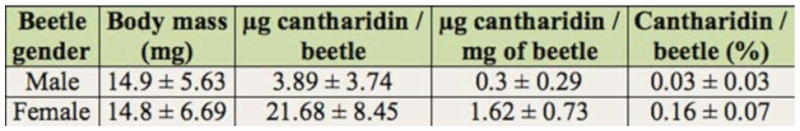
Cantharidin content in studied oedemerid beetles. Values are means ± SD.

**Table 2.**
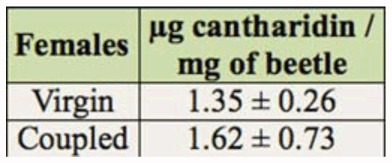
Cantharidin amount in virgin and coupled females.

## Discussion

The main result of this study was the demonstration of cantharidin in *O. p. ventralis* collected from the southern slopes of Mount Elborz, Iran. This subspecies also occurs in south Azerbaijan and southwest Turkmenistan (Svihla 1999). *O. p. ventralis* were found to have 0.03–0.16% of their weight be cantharidin. This percentage of body weight is lower than that in previously studied blister beetles in Iran (0.2–6% of beetle weight) (Nikbakhtzadeh and Tirgari 2002), but it is still sufficient to irritate human skin (Carrel 1986).

Previous studies of cantharidin content of three other species of oedemerids are shown in Table 3. This study quantified cantharidin contents in both virgin and mated female *O. p. ventralis*. There was no significant difference between them (Table 2), showing that both sexes can produce cantharidin. This was previously shown in another oedemerid beetle, *Oedemara femorata* (Holz and Strell 1994). Oedemerid beetles are unique in that both sexes are able to produce cantharidin, whereas only male *O. p. ventralis* produce cantharidin, transferring it to females during copulation (Capinera 2008; Mullen and Durden 2009). Moreover, the amount of cantharidin in female *O. p. ventralis*, even in virgin ones, is 5–6 fold more than males. This result supports the hypothesis that females produce cantharidin. The higher content of this chemical has been attributed to achieving a greater degree of protection for survival of their progeny (Capinera 2008; Mullen and Durden 2009).

**Table 3.**
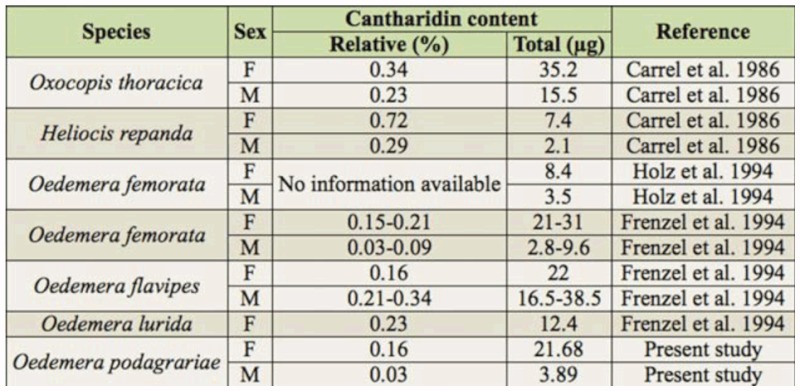
Cantharidin content in oedemerid beetles in both other studies and the current study.
